# Medical Opinion and Movement

**Published:** 1911-03-04

**Authors:** 


### Medical Opinion and Movement.

### Calcified Tubercular Foci.

It is generally recognised that foci of calcification in the lungs and lymphatic glands indicate a previous tubercular affection. Recent research has tended to show that the tubercle bacilli are not completely destroyed by this process of calcification. A series of researches has just been completed by Dr. Wegelin at the Pathological Institute of Bern which confirms this view. He divides these foci into two categories, according as they are calcified or only chalky. The chalky deposits are much softer and more easily cut in sections. In ten cases of calcified deposits he found the bacilli present in four, but of the three cases examined of chalky deposits tubercle bacilli were present in each one. The bacilli were very few in number, requiring much care to detect their presence. Schmitz, who has also investigated this subject, suggests that the bacilli encased in these deposits may in certain circumstances become liberated and again set up a tubercular infection in the organism. This might happen, for instance, in the course of an infectious disease by an œdema and inflammatory swelling of the surrounding tissue. Dr. Wegelin, however, thinks such an event only probable in the case of the softer chalky deposits. Moreover, the number of bacilli present is so small, and their virulence so attenuated, that their liberation would probably not be of serious import. Experimental research goes to show that to produce a tuberculosis the organism must be invaded by a certain minimum quantity of tubercle bacilli.

### Saline and Adrenalin Injections.

Intravenous injections of normal saline with a few drops of 1 in 1,000 adrenalin solution have gained so much in favour recently as a means of combating post-operative shock in abdominal surgery, that it is desirable to call attention to certain dangers attending the treatment, as evidenced by some cases reported in German current medical literature. Dr. J. Berendes, of Schöneberg, reports two cases in which the intravenous injection of 1 litre of normal saline with 8 per cent. glucose and 8 minims of 1 in 1,000 adrenalin solution was followed immediately by rigors and a rise of temperature to 39°.5 and 40° C. The one patient was a woman who had undergone resection for gastric ulcer, and the other was a case of colotomy for obstruction. In both cases the temperature fell to normal in the course of a few hours. A third case reported by Dr. W. Merkens, of Oldenburg, was that of a man aged fifty, obese and diabetic, who was operated upon for purulent peritonitis following perforated appendix. The following day the abdomen was somewhat distended, temperature about 38° C., pulse 130 to 140. One litre of normal saline with 10 minims of adrenalin solution was injected intravenously. Immediately afterwards the patient became pale and distressed, and then had a violent rigor and became unconscious. The pulse became excessively rapid and then imperceptible, and the patient died a few hours later. Yet another case is recorded by Dr. C. Häberlin, of Nauheim. The patient was a woman, aged twenty-five, operated upon for purulent peritonitis following perforation of the appendix. Three day afterwards, the condition having become serious, an intravenous injection of 1½ litre of normal saline with 8 minims of freshly prepared adrenalin solution was made. An hour afterwards the patient had a severe rigor, the pulse accelerated to 160, and the temperature rose to 39°.5 C. The patient died fourteen hours later.

### Purpura Hæmorrhagica.

Bacteriological research has shown such a variety of micro-organisms present in conditions of purpura hæmorrhagica that the theory of a specific microbe being the causal agent of this morbid state has been abandoned, and it has been supposed that even in the infectious purpuræ a process of intoxication is the chief factor. It is further generally recognised that a certain predisposition on the part of the individual or the presence of certain organic lesions, either gastro-intestinal, hepatic, renal, or nervous, favour the appearance of the condition. But, according to the researches of Dr. Di Giuseppe, there is invariably some affection of the suprarenal capsules present in these cases. He thinks that this fact has hitherto escaped notice, because so little attention has been given to these organs till recent years that they have not been taken into consideration in cases of purpura hæmorrhagica that have been submitted to post-mortem examination. The author has carried out a series of experimental observations on animals by which he finds that after inducing a lesion of the suprarenals, either mechanically or chemically, any pathogenic microbe introduced into the circulation will determine a purpura varying in intensity according to the extent of injury to the suprarenals and the virulence of the micro-organism introduced.

### Infantile Gastro-enteritis.

Dr. P. Bonnier has already claimed to be able to effect a cure in many cases of digestive disturbances in adults by light galvano-cauterisation of the nasal mucous membrane. The treatment is based upon the hypothesis that by this cauterisation of the nasal mucous membrane over the inferior turbinated bones, the bulbar nerve-centres of the digestive apparatus are directly affected. At a meeting of the Société de Biologie, Dr. Bonnier made a further communication, in which he claims to obtain even better results by this treatment in infants. In seven infants of less than a year old suffering from intestinal troubles he has obtained a complete cure within twenty-four hours after cauterisation. In another case, aged four months, and suffering from diarrhœa with green stools, which resisted other medical treatment, the stools improved within a few hours of the first cauterisation, and the diarrhœa disappeared and the child steadily put on weight. Yet another infant, aged five weeks, *in extremis*, recovered twenty hours after cauterisation, and took the breast vigorously. The improvement continued, and the child is now in excellent health.

### A New Diabetic Food.

According to Friedenwald and Ruhräh, the soya bean is likely to prove valuable as a food for diabetics. This product is at present grown mainly in Manchuria and Japan; from the former country especially large cargoes of it are exported to Britain for use in the manufacture of oil-cake cattle-food. It is also grown in the United States, but is not used there as food for man. In Japan it is consumed by mankind, and it is suggested that its very high nitrogenous content accounts for the sparing meat diet of the people. Not only is the protein content remarkably high; the quantity of starch is very small, extraordinarily so compared with all other beans to which it is closely related. The beans are not entirely unfamiliar as comestibles, for they are a constituent of many sauces. For this new purpose, as suggested by these authors in the *American Journal of the Medical Sciences*, they can be taken as vegetables after boiling; or made into gruel after being ground into flour; or mixed with a small quantity of wheat flour and baked into biscuits; or served as salad; or turned into thick soup. After feeding diabetic patients on the soya bean the authors have in some cases found such a rapid diminution of sugar in the urine that they speak of the possibility of some active therapeutic action in addition to its dietetic properties.

### Chronic Benzol Poisoning.

Acute poisoning by the fumes of benzol is a long recognised danger to be guarded against in those industries and occupations where benzol is employed. The history of four cases investigated by Mr. C. Glaser, and communicated to the *Johns Hopkins Hospital Bulletin* by him, shows that chronic poisoning from the same source is a very serious possibility, for two of the patients died. Analysis of the urine in two cases showed the presence of small quantities of nitrobenzol; and in the brain of one of the fatal cases the same substance was detected. Both in the urines and the brain aniline also was found. The author ascribes the sickness and deaths to aniline, absorbed during long periods in the shape of nitrobenzol and accumulated in the body until symptoms of poisoning ensued. It is pointed out that chemical methods of great delicacy are required to detect nitrobenzol in the small quantities in which it was here found—hence, the author believes, some failures to find it in previous cases. Analyses of the C.P. benzol (commercially pure) used in the canning factory whence the patients came showed that both aniline and nitrobenzol were present in some drums, but not in others. The standard drum, for instance, by which the contract was made, contained no nitrobenzol and only the faintest trace of aniline; others contained no aniline, but perceptible quantities of nitrobenzol, and others contained traces of both. The tests show that C.P. benzol, free from nitrobenzol, can be, and at times is, furnished.

### An Improvement in Gastro-enterostomy.

Regurgitation from the duodenum into the stomach is not very uncommon after gastro-enterostomy, and in some patients causes great discomfort. Many devices have been employed to avert it, but none of them with entire success. In the *International Journal of Surgery* Dr. Johnson describes a new technique invented by himself with this object. He makes a horseshoe-shaped incision in the stomach, with the convexity downwards (apparently this is on the anterior wall of the stomach). The width of the horseshoe is slightly greater than that of the incision in the duodenum, which is made in the usual manner. The free edge of the flap of stomach is then sewn all round with an over-and-over stitch, the mucosa being thus fixed to the serous coat at their respective edges. Then the stomach and duodenum are united as in the classical operation. The upper edge of the duodenal opening is stitched to the concave edge of the aperture in the stomach; then the lower edge of the duodenum is fixed to the sides of the horseshoe until it reaches the ends of the shoe, where the incision stops; the suturing is then continued around and above the upper part or base of the flap. Lembert's sutures are then used to bury the whole line of junction. Thus the flap of stomach wall hangs free in the duodenum and acts as a valve which permits the passage of food, etc., from the stomach, but closes against any gas or liquid which attempts to pass from the duodenum. The results are said to be excellent.

### Molluscum fibrosum gravidosum.

In the year 1906 Brickner described a variety of molluscum fibrosum which is peculiar to pregnancy. A somewhat different variety of molluscum associated with pregnancy is now described in the *American Journal of Obstetrics and Gynæcology* by Dr. B. C. Hirst; it appears to be a condition not hitherto recognised. At the age of eighteen his patient noticed four or five patches of ordinary molluscum fibrosum on the anterior surface of the body. They remained stationary, and did not increase in number. At twenty-five she married, and a year later became pregnant. The growths rapidly increased in number up to the date of delivery; after this their growth ceased, but those already developed remained. In each successive pregnancy there has been an increase in the number of growths, but no such increase in the interval between the pregnancies. The photograph of the present condition shows the body covered with hundreds of these growths. The case differs from that reported by Brickner in the absence of pigmentation, the failure of the growths to disappear after delivery, and in the greater size and number of them. In this case there was a tendency to molluscum before marriage, whereas in Brickner's patient the growths appeared for the first time during pregnancy. Dr. Stelwagon has pronounced the case to be unique in his own experience and in the literature.

### Sulphate of Magnesium Intravenously.

After some experimental work upon animals, Dr. Huggins has tried the intravenous injection of magnesium sulphate for bacteriæmia in six cases of puerperal sepsis; the results have been so encouraging that he publishes a brief record of them in the *American Journal of Obstetrics and Gynæcology*. Curiously enough, magnesium sulphate seems to have little effect on streptococci *in vitro*; even a 1 per cent. solution does not inhibit the growth of the streptococci. In rabbits the solution when injected into the veins causes inhibition of respiration, and also produces anæsthesia, which supervenes without any preceding stage of excitement. Dr. Huggins' patients received 30 grains of the salt in 8 ounces of normal saline, delivered slowly into a vein at a temperature of 105° to 108° F.; the time occupied in running in this quantity is twenty minutes. If the solution is allowed to flow more rapidly than this, the patient gets embarrassment of breathing and a sensation of heat all over the body. In this manner the author has given fifty injections, without any ill effects. The six cases of puerperal sepsis were all very severely infected: five of them had streptococci in the blood, and the other one had them in the uterus. All six did well, and recovered rapidly and permanently within a comparatively short time after the treatment was exhibited. The charts are published with the article, and show clearly the clinical course of the case. The author recognises that six cases are not sufficient to dogmatise on, but proposes to give the method further trial.

### Rectal Hæmorrhage due to Ectopic Gestation.

A very puzzling case of rectal hæmorrhage was reported to the New York Obstetrical Society in December by Dr. S. M. Brickner. A woman who had not menstruated for eight weeks believed herself pregnant, and attempted to procure abortion by thrusting a bougie into the uterus. She came under care a few days later with a high temperature, an exudate in Douglas' pouch, and with bleeding from the uterus. The exudate increased steadily in size, and presently she began to lose blood per rectum. The latter was explained, apparently, by the finding of a fishbone in the rectum; it was accordingly removed, hæmorrhage ceased, and the patient, against advice, went home. Within three days she began to bleed per rectum again, and after a week of steady loss returned to hospital. Eventually an abdominal section was performed, and it was discovered that the supposed exudate was a ruptured ectopic gestation which had burrowed into the broad ligament and attached itself to the rectum. Although no villi could be seen in the rectum, four inches of which were removed with the adherent mass, there can be little doubt that the chorionic villi must have penetrated right to the mucosa. The patient did well.

### The Underfeeding of the Breast-fed.

An interesting discussion took place at a recent meeting of the Société de Pédiatrie de Paris, with reference to the underfeeding of breast-fed children. Madame Nageotte has studied the question in the Russian colony of the city, and finds that such a state of affairs practically never exists among the children of poor women, who give the breast at all times and without following a properly worked-out plan. On the other hand, among the educated, who suckle their babes every three, or more commonly for their personal convenience, every four hours, underfeeding and its consequences are frequently met with. This state of affairs is gradually getting more common. Dr. Variot, who followed, accused the accoucheurs of being responsible for this underfeeding in that they had made their rules for feeding too inelastic. They seemed to forget that both the quality and the quantity of the milk must be taken into consideration when prescribing rules. Dr. Guinon believed that the movement for the regulation of feeding, with consequent hypo-alimentation, was a European and, in fact, a world one. It was a difficult thing to settle the exact dose of milk to give to a child, since temperament and hereditary antecedents had to be taken into account. It would not be fair to compare the rations given to a healthy little Russian with those allowed a small Parisian, often the issue of debilitated or even tainted parents.

### The Offspring of Tuberculous Mothers.

Vittoz, in a "Thèse de Lyon," recounts the researches he has prosecuted with regard to the offspring of tuberculous mothers, and their chance of surviving after birth. He states that his figures show that 68 per cent. of these children die almost at once, and that many of the remainder are in a deplorable condition and run the risk of a rapid infection considering the contaminated and unhygienic surroundings to which they are subjected. Consequently he believes that, when there is a question of putting an end to a gestation in the interest of the mother, the argument drawn from the possible survival of a child if the mother be allowed to go to term has but a relative value.

### Vaccination against Typhoid.

At a recent meeting of the Académie de Médecine de Paris, Dr. Vincent gave an interesting report on antityphoid vaccination with regard to the methods employed and the results obtained in France, England, Germany, Russia, and the United States. He is led to the conclusion that the process is a rational and practical one, and that it diminishes the frequency and the gravity of typhoid fever in quite a number of cases. He recommends it particularly to all those who by their employment or by reason of some temporary necessity are exposed to direct or indirect infection with the bacillus of typhoid, such as soldiers, sailors, doctors, students, male or female nurses, young people coming from the country into infected towns, and persons in families in which a case of typhoid fever has occurred.

